# Insights on the Use of Transgenic Mice Models in Alzheimer’s Disease Research

**DOI:** 10.3390/ijms25052805

**Published:** 2024-02-28

**Authors:** Mafalda Soares Pádua, José L. Guil-Guerrero, José A. M. Prates, Paula Alexandra Lopes

**Affiliations:** 1 CIISA—Centro de Investigação Interdisciplinar em Sanidade Animal, Faculdade de Medicina Veterinária, Universidade de Lisboa, 1300-477 Lisbon, Portugal; mafaldapadua@fmv.ulisboa.pt (M.S.P.); japrates@fmv.ulisboa.pt (J.A.M.P.); 2 Laboratório Associado para Ciência Animal e Veterinária (AL4AnimalS), Faculdade de Medicina Veterinária, Universidade de Lisboa, 1300-477 Lisbon, Portugal; 3 Departamento de Tecnología de Alimentos, Universidad de Almería, 04120 Almería, Spain; jlguil@ual.es

**Keywords:** Alzheimer’s disease, amyloid precursor protein, neurofibrillary tangles, tau protein, transgenic mice, neurodegenerative disease

## Abstract

Alzheimer’s disease (AD), the leading cause of dementia, presents a significant global health challenge with no known cure to date. Central to our understanding of AD pathogenesis is the β-amyloid cascade hypothesis, which underlies drug research and discovery efforts. Despite extensive studies, no animal models of AD have completely validated this hypothesis. Effective AD models are essential for accurately replicating key pathological features of the disease, notably the formation of β-amyloid plaques and neurofibrillary tangles. These pathological markers are primarily driven by mutations in the amyloid precursor protein (APP) and presenilin 1 (PS1) genes in familial AD (FAD) and by tau protein mutations for the tangle pathology. Transgenic mice models have been instrumental in AD research, heavily relying on the overexpression of mutated APP genes to simulate disease conditions. However, these models do not entirely replicate the human condition of AD. This review aims to provide a comprehensive evaluation of the historical and ongoing research efforts in AD, particularly through the use of transgenic mice models. It is focused on the benefits gathered from these transgenic mice models in understanding β-amyloid toxicity and the broader biological underpinnings of AD. Additionally, the review critically assesses the application of these models in the preclinical testing of new therapeutic interventions, highlighting the gap between animal models and human clinical realities. This analysis underscores the need for refinement in AD research methodologies to bridge this gap and enhance the translational value of preclinical studies.

## 1. Introduction

Over the last decades, a rapid demographic ageing of the population has occurred and is still in progress. The World Health Organization (WHO) estimates that by 2050, the population over the age of 60 will massively increase, reaching a total of 2.1 billion people [1,2]. In Europe, during the last two decades, the percentage of the population aged 65 years old and above increased by 5%, going from 16% to 21%. Portugal alongside Italy are the European countries with the highest shares, accounting for approximately 24% of the aged population [3].

Ageing is the progressive accumulation of changes with time that are associated with or responsible for an ever-increasing susceptibility to disease and death [4]. Human ageing leads to the development of cognitive and physical impairments and consequently a much higher susceptibility to develop dementia and associated neurodegenerative diseases [5,6]. According to the WHO [7], the number of people living with dementia worldwide, a syndrome induced by brain disturbances that affect memory, behaviour and ultimately the capacity to perform daily routine tasks, has been growing quickly and is predicted to rise from 55 million in 2019 to 139 million until 2050 [1,7,8]. Correspondingly, Portugal is of major concern to these emerging circumstances since dementia prevalence has been reported to increase in the latest epidemiological studies [9,10].

### 1.1. Alzheimer’s Disease Pathology

Alzheimer’s disease (AD) is the most common cause of dementia and is a neurological multifactorial disorder described by synaptic damage and neuronal loss, impacting mostly the cortex and hippocampus [11,12]. Patients with this disorder exhibit consistent β-amyloid plaques and neurofibrillary tangles in their brain, turning these features into the pathological hallmark features of AD [11,13,14]. β-amyloid plaques are depositions of oligomers of this peptide, caused by an imbalanced cleavage of the amyloid precursor protein (APP) by α-, β- and γ-secretases and/or decreased degradation of β-amyloid, which, because of their innate misfolded nature, expose several chemical groups qualifying them as toxic [12,14,15,16,17,18,19], while neurofibrillary tangles are hyperphosphorylated forms of the microtubule-associated tau protein resulting from the imbalanced activity of phosphatases and protein kinases, which aggregate and settle in the neuron’s body and dendrites, entirely disrupting its structure and function [12,14,17,18,20].

Despite being considered a major public health concern and the fact that exceptional advances have been made towards Alzheimer’s understanding, up until now, no effective treatments exist to cure patients from AD [8,21]. Therefore, the investigation of AD, focusing on underlying physiological processes as well as progress on its early detection and therapy, stands now as a research priority.

### 1.2. Alzheimer’s Disease Research Models and Limitations

Since conducting experimental surveys on AD directly in humans is unethical and impractical, due to the inaccessibility of the human brain, apart from the post-mortem analysis in which most of the physiological systems are no longer active, considerable experimental disease models have been developed to give further insights into the onset and progression of AD pathophysiological aspects as well as to develop new potential diagnostic and therapeutic strategies, comprising also preclinical testing [18,22,23,24].

None of the existing models so far have completely described human AD, in its pathological, biochemical and behavioural features. Since there is no animal that spontaneously develops both AD hallmarks (β-amyloid accumulation and tau hyperphosphorylation), the most accepted models for the study of this disease are in vitro 2D and 3D cell cultures as well as in vivo transgenic animal models [22,23,25,26,27].

Two-dimensional and three-dimensional cultures comprise brain cells or stem cells (embryonic or induced pluripotent) from patients that will ultimately differentiate into several somatic cells, such as the ones existing in the human brain (e.g., neurons, astrocytes, microglia, etc.) and are cultured to mimic some innate features in native human brain [23,28]. However, these models have several limitations, such as the insufficient availability of tissue from AD patients along with epigenetic and/or phenotypical variations in cellular lines derived from the donors [18,23,25,29]; long-term and expensive cultures, without standardized protocols, to obtain aged brain conditions associated with this neurodegenerative disease [18,23,27,28,29,30,31]; technical difficulties associated with the reconstruction of the most affected brain areas, such as the hippocampus and specific cortical layers and also on performing imaging studies [18,31,32]; and finally unattainable representation of the complex environment found in the human brain, which should accommodate a bigger diversity of important brain cell types, complex cell–cell interactions (between neurons from all around the brain and other cell types that integrate it), and vascular and drainage systems [23,25,27,28,31]. Another important aspect that will never be possible to reach with these models is behaviour assessment, thus limiting these models to early stages of drug development [24,33].

Even though some limitations have been overcome by the progression from 2D cultures to advanced 3D cultures, some of them still prevail. Therefore, in this review, we will concentrate our focus mainly on in vivo transgenic animal (Tg) models for AD. Tg models are living organisms in which particular genes, chosen based on human gene linkage studies associated with AD, have been modified/introduced into the animal’s genome (e.g., genetic manipulation), producing a model that closely mimics human disease, consequently reducing the need to use primates or even humans [34,35]. These models are of extreme value not only as tools for developing insights into the biological basis of AD, due to their high physiological relevance as well as resemblance to structural and biochemical brain ageing [18,22,27,35], but also as a key role for drug development and therapeutical screening, in preclinical testing, due to the possibility of behavioural examination [18,22,26]. Choosing mice as the animal model has several advantages, such as (1) being one of the smallest mammals with a big genome similar to the human genome while requiring low-cost maintenance regarding housing and feeding facilities, enabling in this way a large scale and high-throughput studies [26,35,36]; (2) them being animals with a short lifespan, allowing feasible long-term surveys associated with aging and neurodegenerative disorders [18,36]; (3) being organisms with better and easier established protocols for genetic manipulation with a wide array of available research reagents, such as the case of antibodies [36,37].

**Figure 1:**
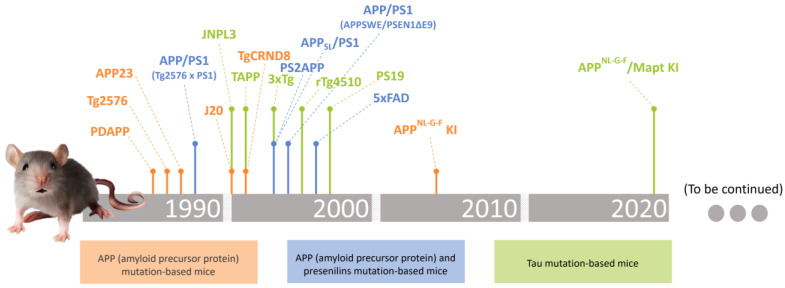
Timeline of transgenic mice models for AD research, according to the literature.

Since the late 1980s, more than 200 mutations associated with AD have been reported and numerous transgenic mice models have originated, which have made substantial contributions [38]. Depending on the expressed mutation, the animal will display distinct clusters of pathological and behavioural features. The desire to amplify these clusters have led to the development of more and more transgenic models, which eventually have become multi-transgenic models [38,39]. In this review, we will describe some of the most conventional and well-characterized mice models developed to date (Figure 1), highlighting the cluster of AD characteristics portrayed for each transgenic experimental model, and enclosing their advantages and disadvantages.

## 2. Transgenic Mice Models in Alzheimer’s Disease Research

### 2.1. APP (Amyloid Precursor Protein) Mutation-Based Mice

These models are based on one of the main AD hallmarks, which is the formation of amyloid deposits in the extracellular regions of the brain. Many of these models are established on the transgenic overexpression of human amyloid precursor protein (APP), combined with different familial AD-associated mutations in the APP gene, resulting in the production of different amyloid peptides that aggregate more easily [40,41,42]. The overexpression of APP was the selected method to mimic β-amyloidosis typical of AD brains generating high β-amyloid values; however, it will additionally produce increased levels of APP fragments, such as sAPP, CTF-α, CTF-β, and AICD, which cause some undesirable effects [40,41,42]. Later on, a new generation of APP mutation-based mice models (knock-in mice) emerged to overcome this problem, in which mice exhibited high values of β-amyloid but without APP overexpression and its consequent side effects [43,44]. Since the 1990s, several APP mutation-based models have been described for AD study based on distinct mutations within the APP gene with different promoters, individually portraying different components of the disease as well as different expression times (Table 1).

#### 2.1.1. PDAPP Model

The PDAPP model was the first AD mouse model developed, overexpressing the human APP gene with the Indiana mutation (V717F) found in the early 1990s, in which a valine is substituted by a phenylalanine at the residue 717, using the PDGF-β promoter [45,46]. This model begins to develop extracellular β-amyloid deposits at approximately 6–9 months of age, which increase as the animal ages, showing an age-dependent progression with regional specificity in the brain similar to AD patients [46]. Alongside this, PDAPP mice also show dystrophic neurites, a loss of synaptic and dendritic density, and increased gliosis [46]. Regarding behavioural disturbances, these mice display acute deficits in a radial maze test from 3 months old as well as reduced object-recognition performance, which progresses in an age-dependent manner and is thought to be associated with memory impairment [47].

#### 2.1.2. Tg2576 Model

Tg2576 is the second developed and one of the most commonly used AD mouse models, overexpressing the human APP695 gene with the Swedish mutation (KM670/671NL) found in the early 1990s. In this model, a lysine is substituted by an asparagine at the residue 670, and a methionine is substituted by a leucine at the residue 671, using the hamster prion promoter [48,49]. This model displays a significant increase in β-amyloid deposits around 11–13 months of age compared to age-matched controls with a similar distribution pattern [49], as reported by Games et al. [46]. In parallel, Tg2576 mice also exhibit dystrophic neurites, a loss of synaptic and dendritic density, and activation of microglial-mediated inflammatory response in and around β-amyloid plaques, with enlargement of its participants [49,50]. Relatively to behavioural disturbances, these mice manifest learning and memory impairment around 9 months old with age-dependent aggravation, assessed by Y and water maze tests [49].

#### 2.1.3. APP23 Model

APP23 mice overexpress the human APP751 gene with the Swedish mutation (KM670/671NL) using the murine Thy1 promoter [51]. This model starts to develop β-amyloid deposits from 6 months of age, increasing its size and number as the animal gets older, predominantly in the neocortex and the hippocampus regions [51]. Moreover, these mice also express dystrophic neurites, glial response in which microglia and astrocytes are detected in β-amyloid plaque surroundings, and for the first-time neuronal loss around 14 months old [51,52]. The density of pyramidal neurons in the CA1 region of the hippocampus is severely reduced as well as in the piriform and entorhinal cortices; however, this is without similar alterations in the neocortex region [52]. Concerning behavioural disturbances, APP23 mice demonstrate cognitive decline with major learning and memory deficits from the age of 3 months old with ageing-related progression, even prior to β-amyloid deposition assessed by a Morris-type water maze test, along with perturbations in circadian rhythms and activities, which are concomitantly observed in Alzheimer’s patients [53,54].

#### 2.1.4. J20 Model

J20 mice overexpress the human APP gene with the combination of two known mutations, the Swedish mutation (KM670/671NL) and the Indiana mutation (v717F), using the PDGF-β promoter [55]. This model starts to display diffuse β-amyloid deposition from 3 months of age; however, J20 mice only present plaque formation around 7–9 months old, predominantly in the hippocampus region, demonstrating age-dependent expression of these peptides [55,56]. Beyond that, these mice also exhibit neuronal loss from the hippocampal CA1 region promptly at 3 months of age, with accentuated progression through ageing, accompanied by inflammation with astrogliosis and microgliosis peak at 6 months old [56]. With respect to behavioural disturbances, J20 mice show hyperactivity and spatial memory deficits from 4 months old, as proven by a radial arm maze test, which became worse as age progressed [56,57]. This model helped to discard conclusions about the neurodegeneration process being dependent on β-amyloid plaque deposition, since neuronal loss, inflammation and behavioural impairment all started well before β-amyloid plaque formation [56].

#### 2.1.5. TgCRND8 Model

TgCRND8 mice overexpress the human APP695 gene encoding both the Swedish (KM670/671NL) and the Indiana (V717F) mutation using the hamster PrP gene promoter [58]. This model displays very rapidly diffuse β-amyloid deposition as well as β-amyloid plaques, being observed as early as 3 months old and quickly progressing with age [58,59]. Simultaneously, these mice exhibit dystrophic neurites encircling plaques together with a focal and prompt inflammatory response with microglial cells, shortly followed by astrocytic gliosis, plus some neuronal loss in the stratum pyramidale of CA1 hippocampal area [58,59,60]. As for behavioural disturbances, TgCRND8 mice sustain spatial learning and memory impairment starting alongside β-amyloid plaque development at 3 months of age, which were assessed by water maze and object recognition tests, as well as deficits in sustained attention, also documented in Alzheimer’s patients [58,61].

#### 2.1.6. App^NL-G-F^ Knock-In Mice Model

App^NL-G-F^ Knock-In mice, unlike the others, express the mouse APP gene in which the β-amyloid sequence was humanized to avoid overexpressing the human APP gene [43]. Like the human APP gene, this mouse APP gene also integrates mutations, such as the Swedish mutation (KM670/671NL), the arctic mutation (E693G) found in the early 2000s, where at the residue 693 (which is within the β-amyloid region of APP) a glutamic acid is replaced by a glycine, and the Iberian mutation (I716F) found in the early 2010s, where at the residue 716 an isoleucine is replaced by a phenylalanine [43,62,63]. This model evidences aggressive β-amyloidosis, with cortical β-amyloid deposition being detected by the age of 2 months old, reaching almost saturation by 7 months old despite plaques being reported only at the age of 6 months old, and their density increasing until 9 months old [43,64,65]. Other than that, synaptic impairment is found in the prefrontal cortex at 3–4 months old, aggravating in an age-dependent manner with extension to the hippocampus at 6–8 months of age [65]. Microgliosis and astrocytosis, as neuroinflammatory responses, are detected from 6 months old [64,66]. Regarding behavioural disturbances, App^NL-G-F^ Knock-In mice reveal some learning and memory impairment from the age of 6–9 months with an age-dependent progression, which was assessed by the Morris water maze, Y maze, Barnes maze, object recognition test and fear conditioning test, as well as anxiolytic-like behaviour detected by the elevated plus maze test from 6 months of age [43,64,67].

**Table 1:** Description of APP (amyloid precursor protein) mutation-based mice models.

APP Mice Model	β-Amyloid Deposits (Onset)	Neurofibrillary Tangles (Onset)	Neuroinflammation	Neuronal Loss	Behavioural Impairments (Onset)	References
PDAPP	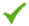 (6–9 months)	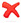	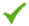	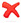	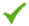 (3 months)	[46,47]
Tg2576	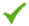 (11–13 months)	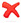	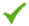	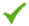	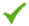 (9 months)	[49,50]
APP23	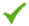 (6 months)	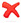	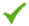	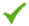	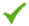 (3 months)	[51,52,53,54]
J20	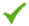 (7–9 months)	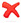	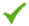	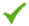	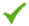 (4 months)	[55,56,57]
TgCRND8	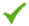 (3 months)	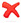	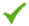	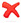	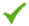 (3 months)	[58,59,60,61]
APP^NL-G-F^ Knock-In	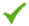 (2 months)	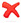	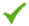	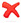	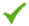 (6–9 months)	[43,64,65,66,67]

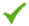
 existing 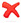 absent.

### 2.2. APP (Amyloid Precursor Protein) and Presenilins Mutation-Based Mice

APP-Tg mice cover some of the classical AD pathological features; however, there are still other components of this disease missing. Therefore, a combination of the previously described AD models with other familial AD-associated mutations was made in an attempt to reach an AD model that can produce the remaining pathological characteristics. Presenilin genes (PS-1 and PS-2) are genes which can carry different familial AD-associated mutations and encode for proteins that are key components of the γ-secretase complex, involved in the cleavage of amyloid precursor protein, among many other cell functions [40,41,42,68]. Mutations in these genes usually result in a shift of secretase cleavage, which consequently produces more amyloidogenic β-amyloid, accelerating β-amyloid deposition, neuronal loss, and behavioural impairment [40,42,68]. Several APP/PS mutation-based models have been described for AD study since the late 1990s and early 2000s, also including Knock-In models, and are based on different combinations of mutations within the APP gene and PS genes, using distinct promoters and individually showing different AD hallmarks as well as different times of disease manifestation (Table 2).

#### 2.2.1. APP/PS1 (Tg2576 × PS1)

APP/PS1 (Tg2576 × PS1) was one of the first developed AD mouse models containing mutations both in APP and PS genes. This animal model results from an interbreed between Tg2576 mice which overexpress the human APP gene with the Swedish mutation (KM670/671NL) and mutant PS1 mice that conceal the M146L mutation in the PS1 gene, found in the mid-1990s, where at the residue 146 a methionine is replaced by a leucine [69,70]. This model starts to display increased concentrations of β-amyloid from the age of 3–4 months old, with a substantial increment around the age of 6 months old when β-amyloid plaques are established in the cortex and hippocampus, progressing further with age [70,71]. In parallel, these mice show activated microglia surrounding β-amyloid deposition from the moment they are detectable (around 3 months of age) and clusters of reactive astrocytes surrounding those same deposits at the age of 6–7 months old [70,71], all together promoting synaptic loss, rather than neuronal loss [72]. Regarding behavioural disturbances, APP/PS1 mice manifest progressive cognitive impairment starting from 3–6 months old, which was assessed by Y-maze, water maze, and radial arm water maze tests [70,73].

#### 2.2.2. APP/PS1 (APP_SWE_/PSEN1ΔE9) Model

APP_SWE_/PSEN1ΔE9 is one of the most used and best-described AD mouse models containing both APP and PS mutations. Mice from this model simultaneously carry the human APP gene comprising the Swedish mutation (KM670/671NL) and the PSEN-1 gene, in which the exon 9 is deleted due to a nonsense mutation in the splice acceptor site of this exon (guanine for thymine) that ultimately does not allow the exon translation, found in the mid-1990’s [74,75]. This model starts to develop β-amyloid plaques in the cortical and hippocampal areas around 4 months old with a progressive age-dependent accumulation [75,76,77,78,79]. Simultaneously, these mice also exhibit a loss of synaptic function with neuronal loss as well as reactive gliosis with astrocytes and activated microglia surrounding β-amyloid plaques in the cortex and the hippocampus [77,78,80,81]. Respecting behavioural disturbances, APP_SWE_/PSEN1ΔE9 mice sustain learning and memory impairment associated with deficits in spatial memory and cognitive flexibility from the age of 8 months old, which was assessed by Barnes maze and Morris water maze tests [79,82,83].

#### 2.2.3. APP_SL_/PS1

APP_SL_/PS1 mice result from a cross between mice expressing the human PS1 gene with the M146L mutation and mice overexpressing the human APP gene bearing the Swedish mutation (KM670/671NL) and the London mutation (V717I), found in the early 1990’s, where at the residue 717 a valine is replaced by an isoleucine [84,85]. This animal model presents an onset of β-amyloid deposits as early as 2.5 months of age, starting small and primarily in the subiculum region of the hippocampus but rapidly increasing in size as animal age progresses, creating plaques and spreading throughout all the regions of the hippocampus and the cortex [85,86]. Alongside, neuroinflammation is found in these mice since 3 months old, initially appearing as clusters of activated microglia together with reactive astrocytes which surround and invade β-amyloid deposits, but ultimately and as age progresses, spreading all around [86,87,88]. Also, synaptic damage and neuronal loss are found at an early age, around 4–6 months old, and have an age-dependent evolution [85,86,89]. Respecting behavioural disturbances and despite studies being scarce in this model, APP_SL_/PS1 mice evidence cognitive impairment associated with memory decline at 9 months of age, which was assessed by an object recognition test [90].

#### 2.2.4. PS2APP Model

PS2APP mice overexpress the human APP gene with the Swedish mutation (KM670/671NL) alongside the human PSEN2 gene with the N141I mutation, found in the mid-1990s, where at the residue 141 an asparagine is replaced by an isoleucine [91,92]. In this animal model, β-amyloid deposits are first detected around 5–6 months old in the subiculum component of the hippocampus and the frontolateral cortex, severely aggravating at 8 months old, and progressing further in an age-dependent manner [92,93,94]. Concomitantly, these mice manifest dystrophic neurites and a reduction in synaptic function as well as an inflammatory response comprising both reactive microgliosis and astrogliosis, starting at the age of 7–8 months old around plaques and spreading out as time goes by [92,94,95]. Relative to behavioural disturbances, PS2APP mice show some learning and memory deficits emerging at 7–8 months old, which were assessed by Barnes maze, Morris water maze and active avoidance tests [92,95].

#### 2.2.5. APP^SL^PS1 Knock-In Mice Model

APP^SL^PS1 Knock-In mice overexpress the human APP gene bearing the Swedish mutation (KM670/671NL) and the London mutation (V717I) and carry within the mouse endogenous presenilin-1 gene, the M233T/L235P mutations, both found in the mid-late 1990s, where at the residue 233 a methionine is replaced by a threonine and at the residue 235 a leucine is replaced by a proline, respectively [96,97,98]. This animal model displays abundant intracellular β-amyloid deposits as early as 2–3 months old in the C1/2 and subiculum regions of the hippocampus and the cortical neurons, accompanied by synaptic function loss, with only a few, small and diffuse extracellular deposits [98,99,100]. At the age of 6 months old, astrogliosis is found close to intracellular deposits, a considerable number of extracellular β-amyloid deposits are perceived, and massive neuronal loss is detected without being spatially associated with the extracellular deposits; all of these features evolve in an age-dependent manner [98,99,100,101]. As to behavioural disturbances, APP^SL^PS1 Knock-In mice exhibit motor disturbances from 6 months of age, which was assessed by balance and suspension tasks and forced swimming test, as well as learning/memory impairment at the same age, which were assessed by Y-maze and T-maze tests [102].

#### 2.2.6. 5xFAD Model

5xFAD mice are currently one of the most widely used AD models, and therefore one of the best characterized, co-expressing simultaneously five FAD mutations, in which three mutations are in the APP gene and the other two mutations are in the PS1 gene. This animal model overexpresses the human APP gene with the Swedish (KM670/671NL), London (V717I) and Florida (I716V) mutations, this last one found in the late 1990s, where at the residue 716 an isoleucine is replaced by a valine, and the PS1 gene with the M146L and the L286V mutations, the last one found in the mid-1990s, where at the residue 286 a leucine is replaced by a valine [103,104,105]. These mice display robust amyloid pathology with increased levels of β-amyloid and intraneuronal deposits since the age of 1.5 months and early formation of extracellular deposits from 2 months old, mainly in the subiculum, deep layers of the cortex and frontal cortex with an age-related progression, covering eventually much of the cortex, subiculum and hippocampus regions [105,106,107,108,109]. Neuroinflammation, assessed by reactive astrocytes and microglia, is detected at 2 months old at the same time as extracellular β-amyloid deposits, progressing in an age-dependent manner, with a similar distribution [105,107,108,109]. Dystrophic neurites are disclosed from 3 months of age followed by synaptic transmission impairment at 4 months old that concurs with the beginning of neuronal decline, which is strongly associated with intra- and extracellular β-amyloid deposits and causes massive neuronal loss at the age of 9 months old [105,106,109,110]. Regarding behavioural disturbances, 5xFAD mice are reported to have memory deficits and learning impairment as early as 1–2 months old, which were assessed by the Morris water maze test [111,112]. However, it is more consensual that 5xFAD mice exhibit cognitive impairment from 4 months old, which was assessed by Morris water maze, Y-maze and olfactory H-maze tests [105,107,113].

**Table 2:** Description of APP (amyloid precursor protein) and presenilin mutation-based mouse models.

APP + PS Mice Model	β-Amyloid Deposits (Onset)	Neurofibrillary Tangles (Onset)	Neuroinflammation	Neuronal Loss	Behavioural Impairments (Onset)	References
APP/PS1 (Tg2576 × PS1)	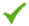 (6 months)	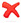	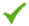	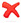	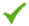 (3–6 months)	[70,71,72,73]
APP/PS1 (APPswe/PSEN1ΔE9)	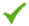 (4 months)	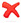	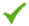	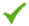	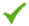 (8 months)	[75,76,77,78,79,80,81,82,83]
APP_SL_/PS1	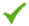 (2.5 months)	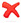	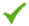	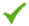	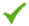 (9 months)	[85,86,87,88,89,90]
PS2APP	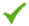 (5–6 months)	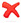	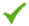	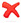	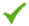 (7–8 months)	[92,93,94,95]
APP_SL_PS1 Knock-In	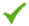 (2–3 months)	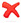	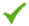	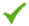	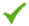 (6 months)	[98,99,100,101,102]
5xFAD	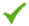 (2 months)	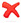	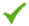	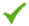	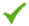 (1–4 months)	[105,106,107,108,109,110,111,112,113]

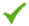
 existing 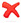 absent.

### 2.3. Tau Gene Mutation-Based Mice

APP and APP/PS mutation-based models sum up several important AD pathological features. However, one of the main hallmarks of this disease is still missing: the neurofibrillary tangles, which are associated with tau pathology. Therefore, mice with mutations in the gene that encodes tau protein, the MAPT (microtubule-associated protein tau) gene, were developed and produced, sometimes in combination with mutations seen priorly, in an attempt to reach an AD mouse model that can completely mimic the AD clinical condition. Tau protein is a microtubule-associated protein present in neurons that modulates the stability of their cytoskeleton due to its central role in microtubule architecture [20,114]. AD-associated mutations in the MAPT gene either alter the amino acid sequence within tau protein—which induces hyperphosphorylation, leads to a reduced predisposition for microtubule assembly, and consequently leads to an increased tendency for aggregation—or imbalance tau’s isoforms due to unusual splicing, also triggering hyperphosphorylation and aggregation [20,114,115,116]. Since the early 2000s, several MAPT mutation-based models have been described for AD research, including Knock-In models, and are based on various mutations within the MAPT gene with the employment of distinct promoters as well as on different combinations with mutations both in the APP gene or in the PS gene, thus individually portraying several AD features as well as different appearance timings (Table 3).

#### 2.3.1. JNPL3 Model

The JNPL3 model was found in the late 1990s and was the first developed AD mouse model carrying an MAPT mutation, which expresses the human 0N4R tau with the P301L mutation that only affects the 4-repeat (4R) tau isoform without changing the isoforms ratio, in which a proline is replaced by a leucine within exon 10, using the mouse prion protein promoter [117,118]. Unlike all the animal models previously described, this model does not develop β-amyloid deposits; however, it forms neurofibrillary tangles (NFT) and inclusion bodies by the age of 4.5 months old in the brainstem and in the spinal cord, progressing in an age-dependent manner, with ‘pre-tangles’ occurring in the cortex, hippocampus, and basal ganglia regions [118,119]. Alongside this, JNPL3 mice also manifest neuronal loss, particularly motor neurons, as well as gliosis with reactive astrocytes and glial filaments, especially occurring in areas where neurofibrillary tangle density is greater [118,119]. As for behavioural disturbances, although these mice evidence a normal cognitive function, assessed by radial arm water maze and Y-maze tests, acute motor and behavioural deficits are clear, with an apparent better behavioural performance until 7 months old assessed by the rotarod performance test, which progressively deteriorates from then on, and by the age of 10 months old major motor impairments are evident, such as hindlimb paralysis [118,120].

#### 2.3.2. PS19 Model

PS19 mice express human 1N4R tau with a P301S mutation, found in the late 1990s, which also affects the 4-repeat (4R) tau isoform without changing the isoforms ratio, in which a proline is replaced by a serine within exon 10, using the mouse prion protein promoter [121,122]. This animal model, like the one before, does not show β-amyloid deposits but develops neurofibrillary tangles and inclusion bodies from 5–6 months of age in the neocortex, hippocampus, brain stem and spinal cord, with an age-dependent progression [122,123]. Synapse loss and neuroinflammation are both detected early on, around 3 months of age, even before neurofibrillary tangles’ occurrence in the hippocampal region, showing an age-dependent evolution [122]. Microglia activation precedes astrogliosis since this last one becomes particularly relevant at 6–8 months old, in parallel with NFT and neuronal loss spread [122,124]. Regarding behavioural disturbances, PS19 mice display motor impairments, such as clasping and limb retraction when lifted by the tail from as early as 3 months old, which progresses to paralysis beginning at 7–10 months old [122]. Moreover, these mice show signs of hyperactivity and reduced anxiety when compared to control groups starting at 3 months of age, and exhibit some learning and memory impairment, assessed by object recognition and Y maze tests at 3 months old, and by Morris water maze test at 8–9 months old [123,124,125].

#### 2.3.3. rTg4510 Model

rTg4510 mice are animals containing the human 0N4R tau with the P301L mutation; however, its expression can be regulated by either suppression with doxycycline or induction with tetracycline–operon-responsive element, using the human CaMKIIα promoter [126,127]. Mice in this experimental animal model develop pre-tangles as early as 2.5 months of age, and tangle-like inclusions by 4 months old in the cortex and by 5.5 months old in the hippocampus, progressing in an age-dependent manner [126,127,128]. Major brain atrophy and neuronal loss are also observed in 5.5-month-old mice, particularly in the hippocampal CA1 and dentate gyrus regions, exhibiting an age-dependent evolution. No changes are observed in motor neurons, contrarily to the previous mice models, as well as no β-amyloid deposits [126,127,128,129]. As to behavioural disturbances, rTg4510 mice evidence cognitive impairment from as early as 2.5–4 months old, mainly associated with age-dependent learning and memory deficits, assessed by the Morris water maze test [126,127,130]. Motor impairments are confirmed by the Morris water maze test or by revealing a grasping reflex when animals are lifted by the tail from 6–10 months of age [126,127]. Notably by using this animal model, it was understood that when human 0N4R tau with the P301L mutation is suppressed by doxycycline, neuronal death and cognitive decline are suspended or even reversed [127].

#### 2.3.4. TAPP Model

The TAPP model results from an interbreed between Tg2576 mice, which overexpress the human APP gene with the Swedish mutation (KM670/671NL), and JNPL3 mice, which express the human 0N4R tau with the P301L mutation, using the mouse prion protein promoter [131]. It is one of the first models to successfully illustrate both AD pathological hallmarks, evidencing the interaction between β-amyloid plaques and tau pathology. This experimental animal model exhibits β-amyloid plaques around 6 months of age which increase in an age-dependent manner, and are similar to Tg2576 mice concerning β-amyloid plaque density and distribution [131,132,133]. In addition, this animal model displays neurofibrillary tangles and reveals an age-dependent progression from as early as 3 months old with identical morphology and distribution to JNPL3 mice [131,132]. Moreover, dystrophic neurites, neuronal loss and gliosis occur from 3–6 months of age, being for the most part associated with β-amyloid plaques [131]. Regarding behavioural disturbances, TAPP mice display motor impairments similar to JNPL3 mice at the same age (10 months old), such as hindlimb weakness and poor performance in rotarod performance test, as well as cognitive impairments associated with learning and memory deficits, assessed by Morris water maze and object recognition tests earlier than 7 months of age [131,132,134].

#### 2.3.5. 3xTg Model

3xTg mice are one of the most used AD models, accommodating three genes with FAD-associated mutations, the human APP gene carrying the Swedish mutation (KM670/671NL), the human MAPT gene carrying the P301L mutation, and the PS1 gene carrying the M146V mutation, using the Thy1.2 promoter and the endogenous PSEN1 promoter [135]. As in the previous TAPP mice, this experimental model evidences both β-amyloid deposits and tau pathology. In these mice, β-amyloid plaques precede the tau pathology, emerging firstly in the frontal cortex and proliferating soon after in an age-dependent manner to the hippocampal region at 6 months old [135,136,137]. Neurofibrillary tangles only appear by 12 months of age, and contrarily to amyloid deposits, firstly occur in the CA1 region of the hippocampus and only after progress to the cortex as the mouse gets older [135,136,137]. Alongside this, synaptic dysfunction and neuroinflammation with activated microglia and reactive astrocytes are perceived from 6 months old, demonstrating an age-dependent increase [136,137]. Regarding behavioural disturbances, 3xTg mice appear to be hypoactive when exposed to open-field tests and manifest age-dependent cognitive decline starting at 6 months old concerning learning and memory, assessed by Morris water maze, Barnes maze, object recognition and passive avoidance learning tests [137,138,139].

#### 2.3.6. APP^NL-G-F^/Mapt Knock-In Mice Model

APP^NL-G-F^/Mapt Knock-In mice, unlike the other mice models that overexpress only one of the six human tau isoforms, have its entire murine MAPT gene humanized without any mutation, using the CaMKIIα promoter, together with the humanized APP gene harbouring the Swedish (KM670/671NL), the Artic (E693G) and the Iberian (I716F) mutations [140]. This experimental animal model evidences amyloid plaques, neuroinflammation, dystrophic neurites and neuronal loss by 6 months old with an age-dependent progression, in all similar to APP^NL-G-F^ Knock-In mice [140,141,142,143]. In addition, from 6 months old, this mouse model also exhibits accumulation of hyperphosphorylated tau in the dystrophic neurites which surround β-amyloid plaques, without neurofibrillary tangles being formed [140,142,143]. As to behavioural disturbances, APP^NL-G-F^/Mapt Knock-In Mice reveal spatial learning decline and memory impairment by the age of 9 months old, assessed by the Morris water maze test [143]. These Knock-In mice demonstrate that it is necessary a mutation in the Mapt gene, even if humanized, to cause tauopathy; notwithstanding, β-amyloidosis by itself leads to an increase in tau phosphorylation.

**Table 3:** Description of tau gene mutation-based mice models.

Tau Mice Model	β-Amyloid Deposits (Onset)	Neurofibrillary Tangles (Onset)	Neuroinflammation	Neuronal Loss	Behavioural Impairments (Onset)	References
JNPL3	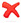	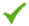 (4.5 months)	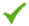	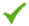	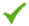 (7–10 months)	[118,119,120]
PS19	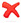	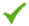 (5–6 months)	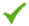	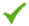	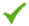 (3–8 months)	[122,123,124,125]
rTg4510	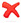	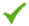 (4 months)	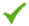	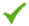	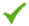 (2.5–4 months)	[126,127,128,129,130]
TAPP	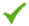 (6 months)	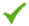 (3 months)	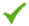	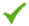	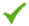 (7–10 months)	[131,132,133,134]
3xTg	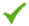 (6 months)	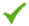 (12 months)	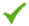	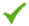	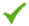 (6 months)	[135,136,137,138,139]
APP^NL-G-F^/Mapt Knock-In	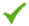 (6 months)	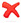	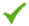	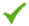	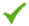 (9 months)	[140,141,142,143]

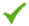
 existing 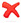 absent.

## 3. Mice Modelling in Alzheimer’s Disease Research: Pros and Cons

Mice are, amongst vertebrates, the most applied model for the study of AD. Transgenic mouse models mimic a wide range of AD-related pathologies, allowing for the recapitulation of Aβ pathology suitable for long-term research and therapeutic screening [18]. There are several reasons for this, including the fact that transgenic technology in mice is moderately low-priced and well-implemented. Moreover, mice have a short life span, are small mammals with minimal dietary cost maintenance and are easily manipulated in animal facilities, under control of strictly environmental conditions, such as room temperature (20–24 °C), lightening (14 h light/10 h dark), ventilation (15 changes/h) or relative humidity (50–60%) [144]. In addition, there is a well-defined behavioural phenotype simply modelled in mice [22]. Transgenic AD rat lines also exist but are not as widely or commercially available as those for mice [145,146,147,148].

On the flip side, transgenic mouse models have been challenged by the difficulty of reproducing the entire spectrum of AD pathology, in particular low dataset reproducibility, and extremely high levels of mutated gene expression to induce pathogenesis, not to mention the variation in features of AD between animal models as well as divergences in AD phenotypes compared to AD human brain [18]. Moreover, various suggestions have been advanced for the difficulty of inducing neurofibrillary tangle-like lesions in the mouse, including the fact that neurofibrillary tangle (NFT) replication needs extra mutations [18], differences between human and mouse tau [32], and also the shorter life span of the mouse. However, the underlying reasons remain unexplained [22].

Relative to behaviour assessment, it is presently unclear if plaque onset and cognitive deficits in mice reflect those of human disease. Many of the mouse models display behavioural deficits before the appearance of relevant plaques [149]. Conversely, substantial plaque pathology is probably present for some time before cognitive symptoms first appear in AD patients [150]. The interpretation of behavioural studies in the mouse is also complicated by the fact that it is difficult to know how precisely behavioural tests mimic cognitive deficits in humans.

## 4. Final Considerations and Future Directions

Whatever its mechanism of action, studies in transgenic mice are sufficiently compelling in defining drug targets and designing novel therapeutic strategies. Regardless of decades of intensive research, the Food and Drug Administration (FDA) agency has approved so far only seven drugs in the treatment of AD. Five of these drugs—donepezil, rivastigmine, galantamine, memantine and memantine combined with donepezil—are aimed at improving symptoms of the disease [151]. Drugs meant at slowing the evolution of the disease have been also recently developed, with the FDA granting accelerated approval for anti-amyloid-β (anti-Aβ) monoclonal antibodies (mabs), particularly for aducanumab on 21 June 2021, and more recently for lecanemab on 22 January 2023 [152]. Anti-Aβ mabs play a role in slowing disease progression in patients with AD, unfortunately at the huge cost of increased probability of negative side effects, such as edema and cerebral microhemorrhages or macrohemorrhages [152]. This number stands alone as indicative of the challenges faced ahead in AD basic investigation, and the key evidence that a large amount of knowledge remains to be disclosed in AD pathogenesis and progression.

In line with this, transcriptomic and proteomic techniques allowed the identification of novel differentially regulated genes and correspondent proteins [23], and have been increasingly applied to animal models of AD, although high maintenance costs are involved. In fact, disease pathogenesis could be better examined using omics approaches that allow genome- or proteome-wide screening for altered networks during disease, and the expanded development of neuroimaging approaches could provide essential information about disease progression in humans [23]. Thus far, proteomic studies in transgenic mouse models indicate that mitochondria, as a cellular entity, is an early target of Aβ and tau aggregates [153]. With advanced imaging techniques that can be used in both humans and mice, an early, preclinical diagnosis of AD could be within reach [153]. Among other expensive imaging techniques, positron emission tomography (PET), computed tomography (CT), magnetic resonance imaging (MRI) and multiphoton imaging are increasingly being applied for the clinical diagnosis of AD [154]. In mouse models, Aβ plaques can be labelled with the PET tracer ^11^C-labelled Pittsburgh Compound-B (PIB), which rapidly enters the brain [153]. Notwithstanding, invertebrate animal models, such as the nematode *Caenorhabditis elegans* and the fruitfly *Drosophila melanogaster*, have been disclosed as a powerful means for AD investigation. In transgenic flies with tau, the neurodegeneration may arise without neurofibrillary tangle occurrence and is usually related to the accumulation of filamentous actin-containing rods [153]. In addition, Sarasa and Pesini [155] have proposed the chick embryo and the dog as better experimental animal models because enzymes involved in the process of human Aβ precursor protein are identical to those of humans. As such, these new species may be also accepted as alternative models to study the biology of AD, and could serve specifically in experiments for Aβ-targeted drugs or novel therapeutic approaches against this disease. Dogs can be of great help to investigate the first stage of AD and to test novel therapies. Dogs in canine models have a fairly lengthy lifespan (12 to 20 years upon dependence of the breed), share owners’ home and environment, and their clinical status as well as typical behaviour are easily to exploit relative to other species. Most determinant, canine APP is very similar (98%) to that from humans, and dogs demonstrate age-dependent brain pathology [155].

In vivo diagnosis of AD is assessed through post-mortem histopathological exam, which most of the times is not feasible due to ethical concerns expressed by families. Post-mortem brains of AD patients display neuropathological features, in particular senile plaques and NFTs, which comprise Aβ peptides and extremely phosphorylated tau protein, correspondingly [156,157].

Besides ethical concerns and legal problems [18], translational alarms with experimental animal models have emerged as serious limitations [158], as follows: (1) there is a clear lack of concordance between preclinical models and human clinical trials which might be associated with non-existence variability among individuals in the models, patients enrolled far too late or existent comorbidities. (2) Conversely to in-bred strains of mice, the participants in clinical trials are too heterogenous. (3) Very early synaptic loss in the majority of rodent models has been reported, indicating that the models translate well the prodromal phase of the disease. The prodromal stage of AD is also referred to as mild cognitive impairment due to AD, and this is the stage where there are obvious symptoms of brain dysfunction. Not only does the individual have to meet criteria for mild cognitive impairment but also the primary underlying pathophysiology is judged by a clinician to be due to AD. (4) models used are of familial AD, and most people have sporadic AD. It is the most common form of Alzheimer’s disease. Sporadic AD has no specific family link and is due to a complex combination of genes, environment and lifestyle. The single greatest risk factor for developing this form of AD is aging.

Current basic research involving animal experimentation has been accused of unreliability and poor translation to human AD [23,159]. Up until now, transgenic mouse models have been troubled by the difficulty of producing the full spectrum of AD pathology. Among other reasons, fundamental species-specific differences in the genome [159], such as dissimilarity of tau gene structures between humans and mice, and protein composition may block the recapitulation of AD pathological events in mouse experimental models. With the incidence of AD anticipated to triple in the forthcoming decades, preventive actions and successful treatments are absolutely warranted. The following decades require an increase in the number of AD patients willing to be enrolled in clinical trials and pursuit research in order to identify standard biomarkers, genetic probability and risk factors, and lifestyle approaches. By focusing the emphasis of AD research on the human patient, it is expected to increase the prospect of identifying novel methods and pharmacological targets through which AD may be definitely prevented, counteracted, and reverted.
